# Current Milestones and Future Horizons in Minimally Invasive Urologic Surgery

**DOI:** 10.3390/jcm13216573

**Published:** 2024-11-01

**Authors:** Benedikt Becker, Clemens M. Rosenbaum

**Affiliations:** 1 Department of Urology, Asklepios Hospital Barmbek, 22307 Hamburg, Germany; c.rosenbaum@asklepios.com; 2 Department of Urology, University Hospital Schleswig-Holstein, Campus Lübeck, Ratzeburger Allee 160, 23562 Lübeck, Germany

## 1. Introduction

Minimally invasive surgery (MIS) has emerged as one of the most significant advancements in the field of urology over the past few decades. The drive toward less invasive methods has been primarily fueled by the desire to reduce perioperative complications, minimize patient morbidity, and enhance recovery times—all while maintaining or even improving therapeutic efficacy. The landscape of urologic surgery has dramatically changed, shifting from open, highly invasive procedures to less traumatic interventions, such as laparoscopic, endoscopic, and robotic-assisted surgeries [1].

Robotic technology in particular has played a pivotal role in this transformation. Since the introduction of robotic-assisted laparoscopic prostatectomy, the adoption of robotic systems has expanded to include a wide range of urologic procedures, demonstrating consistent improvements in precision, ergonomics, and visualization. The use of advanced imaging modalities, such as fluorescence guidance, and the development of more efficient energy delivery tools for lithotripsy have only further accelerated this evolution.

In this Special Issue, we present five pivotal studies that collectively illustrate the ongoing advancements in minimally invasive urologic surgery. These studies explore the novel applications of cutting-edge technologies, ranging from robotic fluorescence-guided surgeries for upper tract interventions to high-intensity focused ultrasound (HIFU) for prostate cancer, as well as a new Thulium laser for kidney stone management. Additionally, they examine critical comparisons, such as laparoscopic versus open adrenalectomy, and identify predictors of complications like ureteral strictures. Each of these studies contributes to the growing body of evidence that supports the efficacy, safety, and future potential of minimally invasive approaches in urology.

## 2. Application of Indocyanine Green in Combination with Da Vinci Xi Robot in Surgeries on the Upper Urinary Tract: A Case Series Study

The first study explores the innovative combination of indocyanine green (ICG) fluorescence with the Da Vinci Xi robotic platform for upper urinary tract surgeries [2]. The integration of ICG fluorescence technology in robotic-assisted urology has proven to be a major step forward in improving surgical outcomes. This case series study highlights its specific use in surgeries like robotic partial nephrectomy and pyeloplasty, where the identification of key anatomical structures is paramount for success.

ICG, administered intravenously, allows for real-time visualization of blood flow, enhancing the surgeon’s ability to distinguish between malignant and normal tissues. This is particularly critical during partial nephrectomies, where the goal is to resect the tumor completely while preserving as much healthy renal parenchyma as possible. The study demonstrated that ICG fluorescence enabled precise identification of segmental renal arteries and improved the delineation of tumor boundaries, thus facilitating more accurate dissection and reducing the risk of positive margins.

Another important aspect discussed in the case series is the role of ICG in preserving critical structures. During reconstructive procedures, such as pyeloplasty, ICG can assist in clearly identifying the renal pelvis and ureter, thereby minimizing the risk of inadvertent injury to the urinary tract or surrounding vessels. The authors also noted that Da Vinci Xi’s advanced optics, when combined with ICG, significantly enhanced the visualization of lymphatic tissues during lymph node dissections, contributing to better oncological resections.

The application of ICG with robotic surgery exemplifies how the merging of multiple advanced technologies can create a synergistic effect that ultimately benefits patient outcomes. By improving the precision of resection, reducing complications, and increasing the overall safety of the procedure, this approach represents a leap forward in complex urologic surgeries.

## 3. Follow-Up of Men Who Have Undergone Focal Therapy for Prostate Cancer with HIFU—A Real-World Experience

The second study focuses on the use of high-intensity focused ultrasound (HIFU) for focal therapy in prostate cancer [3]. This real-world analysis captures outcomes in men who were managed with focal therapy, underscoring its viability as a less invasive treatment option that spares patients from the more significant morbidities associated with radical prostatectomy.

HIFU, as a focal therapy, aims to target only the cancerous regions within the prostate, leaving the surrounding healthy tissue unharmed. This approach results in significantly lower rates of incontinence and sexual dysfunction compared to radical treatments. The study provides valuable insight into patient selection criteria, emphasizing the importance of preoperative imaging with multiparametric MRI to accurately identify candidates with localized, clinically significant disease. The use of MRI fusion-guided biopsy also ensures that treatment is targeted precisely to the areas of highest risk.

The follow-up data in this study show that HIFU offers satisfactory oncological control in the medium term, with acceptable rates of biochemical recurrence. Importantly, the quality-of-life outcomes reported by the patients were encouraging, with most patients maintaining normal urinary and sexual function post-treatment. The study’s real-world context highlights the feasibility of implementing HIFU in diverse healthcare settings and suggests that it could be an effective alternative to more invasive procedures, particularly for men with low-to-intermediate-risk prostate cancer.

Focal therapies like HIFU embody the principles of personalized, patient-centered care, which are at the core of minimally invasive urology. By providing effective cancer control while preserving function, HIFU and similar approaches might represent the future of prostate cancer management, where a balance between oncological safety and quality of life is paramount.

## 4. Laparoscopic or Open Adrenalectomy for Stage I–II Adrenocortical Carcinoma: A Retrospective Study

The retrospective study comparing laparoscopic and open adrenalectomy for Stage I–II adrenocortical carcinoma (ACC) provides critical insights into the evolving management of this aggressive malignancy [4]. Traditionally, ACC has been managed with open surgery due to concerns about the risk of tumor spillage and incomplete resection with minimally invasive techniques. However, recent advancements in laparoscopic and robotic technologies have led to a re-evaluation of these approaches in the treatment of localized ACC.

This study’s findings indicate that laparoscopic adrenalectomy offers comparable oncologic outcomes to open surgery for patients with early-stage ACC. Laparoscopic surgery resulted in reduced intraoperative blood loss, shorter hospital stays, and quicker recovery, which are consistent with the advantages seen in other laparoscopic procedures. Importantly, there were no significant differences in disease-free survival or overall survival between the two groups, suggesting that laparoscopic adrenalectomy is a safe and effective alternative to the open approach for selected patients.

The authors emphasize the importance of surgical expertise, noting that the laparoscopic approach should only be performed by surgeons with extensive experience in minimally invasive adrenal surgery, particularly when managing potentially aggressive tumors like ACC. The study also discusses the potential for robotic-assisted adrenalectomy to overcome some of the limitations of conventional laparoscopy, such as its limited range of motion and the difficulties in dissection in challenging anatomical locations. Robotic systems, with their enhanced dexterity and three-dimensional visualization, may further improve the safety and feasibility of minimally invasive adrenalectomy for ACC in the future.

## 5. First Clinical Experience of a Novel Pulsed Solid-State Thulium Laser During Percutaneous Nephrolithotomy

Percutaneous nephrolithotomy (PCNL) is a well-established treatment for large and complex kidney stones. The fourth study reports on the clinical application of a novel pulsed solid-state Thulium laser during Mini-PCNL, highlighting the potential benefits of this technology in improving the efficiency and safety of lithotripsy [5].

The Thulium laser represents a significant advancement over the traditional Holmium laser, which has been the standard for many years. The new laser technology offers improved energy delivery with a more consistent pulse pattern, allowing for better fragmentation of stones. The authors reported that the Thulium laser provided effective dusting of renal calculi, which minimized the need for basket extraction and reduced overall operative time. Furthermore, the reduced retropulsion effect observed with the Thulium laser allowed for more controlled stone fragmentation, decreasing the risk of injury to the renal parenchyma and ureter.

The study also noted a reduction in intraoperative complications, including hemorrhage and mucosal injury, attributed to the precise control offered by the Thulium laser. The authors suggest that the adoption of this laser technology could lead to shorter procedure times, lower complication rates, and improved stone-free rates, making it a valuable addition to the armamentarium of urologic surgeons. As new laser technologies continue to evolve, their integration into minimally invasive procedures like Mini-PCNL is expected to further improve outcomes for patients with urolithiasis.

## 6. Predictors of Ureteral Strictures After Retrograde Ureteroscopic Treatment of Impacted Ureteral Stones: A Systematic Literature Review

The final study presented in this Special Issue is a systematic review that identifies predictors of ureteral stricture formation following retrograde ureteroscopic treatment of impacted ureteral stones [6]. Stricture formation is a significant complication that can necessitate further interventions, adding to patient morbidity and healthcare costs.

The review highlights several key risk factors for stricture development, including larger stone size, prolonged duration of impaction, and the use of higher laser energy settings during ureteroscopy. These findings underscore the importance of careful preoperative planning and individualized treatment protocols. For example, in patients with large impacted stones, consideration could be given to preoperative stenting or even alternative treatment modalities, such as PCNL, to reduce the risk of ureteral damage.

Additionally, the authors discuss the potential benefits of newer ureteroscopic technologies, such as digital ureteroscopes with improved visualization and the use of ureteral access sheaths, which can help to minimize trauma during stone retrieval. The review calls for the development of standardized protocols to better predict and prevent strictures, emphasizing the importance of minimizing trauma to the ureter during stone manipulation and fragmentation. As the field of endourology continues to advance, it is likely that improvements in both equipment and technique will further reduce the incidence of such complications, enhancing the safety and efficacy of ureteroscopic stone management.

## 7. Conclusions

The studies presented in this Special Issue collectively highlight the remarkable progress that has been made in the field of minimally invasive urologic surgery. The integration of advanced imaging modalities, novel laser technologies, and robotic platforms has allowed for increasingly precise and effective interventions that reduce patient morbidity and enhance recovery.

The trend toward robotic-assisted surgery is evident across the spectrum of urologic procedures, and it is likely that robotic systems will eventually become the standard for most surgical interventions currently performed laparoscopically or openly. The advantages of robotic systems, including superior dexterity, enhanced visualization, and the ability to incorporate adjunct technologies such as fluorescence guidance, have been demonstrated in various studies and are contributing to improved outcomes for patients.

Looking to the future, the integration of artificial intelligence and machine learning into robotic systems holds great promise for further enhancing intraoperative decision-making, reducing the learning curve for complex procedures, and ultimately improving patient outcomes. Furthermore, the ongoing development of cost-effective robotic platforms will make these technologies more accessible, expanding their use to a broader range of healthcare settings and patient populations.

As we continue to push the boundaries of what is possible with minimally invasive surgery, our ultimate goal remains the same: to provide the highest level of care for patients while minimizing the impact of surgical intervention. The studies featured in this issue are a testament to the progress that has been made and the potential that lies ahead. The future of urologic surgery is undoubtedly minimally invasive, with robotics leading the charge toward more precise, effective, and patient-centered care.
